# 

**Published:** 1878-02

**Authors:** 


Whole Number. CCCXCIII.

February, 1878.

Number 2. Vol. XXXVI

THE

### CHICAGO MEDICAL Journal and Examiner

**EDITORS:**

WILLIAM H. BYFORD, A.M., M.D.

JAS. NEVINS HYDE, A.M., M.D. FERD. C. HOTZ, M.D.

E. FLETCHER INGALS, M.D.

CHICAGO:

*PUBLISHED BY THE CHICAGO MEDICAL PRESS ASSOCIATION.*

188 Clark Street.

☞ **Read the Notice of this Journal among Advertisements.**

